# Exposures in adult outpatients with COVID‐19 infection during early community transmission, Tennessee

**DOI:** 10.1111/irv.12792

**Published:** 2020-08-04

**Authors:** Mark W. Tenforde, Leora R. Feldstein, Christopher J. Lindsell, Manish M. Patel, Wesley H. Self, for the IVY Network Investigators, CDC COVID‐19 Response Team, H. Keipp Talbot, Carlos G. Grijalva, Todd W. Rice, Adrienne H. Baughman, Robert McClellan, Li Wang, Kimberly W. Hart, Nathan I. Shapiro, Ahmed M. Kassem, Courtney N. Sciarratta, Nicole Dzuris, Eric P. Griggs, Emily R. Smith, Constance E. Ogokeh, Michael Wu, Sara S. Kim, Paula L. Marcet, Akshita Siddula

**Affiliations:** ^1^ CDC COVID‐19 Response Team Atlanta GA USA; ^2^ Vanderbilt University School of Medicine Nashville TN USA

United States guidelines on social distancing were issued on March 16, 2020, following early community transmission.^1^ To ascertain sources of presumptive exposure risk factors among non‐hospitalized adults with SARS‐CoV‐2 infection in the period coinciding with issuance of national social distancing guidelines, we performed a cross‐sectional telephone survey of adults with SARS‐CoV‐2 infection diagnosed within the Vanderbilt University Medical Center (VUMC) system in Nashville, Tennessee.

We randomly sampled 156 adults from 322 adults ≥18 years who tested positive for SARS‐CoV‐2, using reverse transcriptase polymerase chain reaction (RT‐PCR) on nasopharyngeal specimen collected at an emergency department (ED) or outpatient clinic between March 31, 2020, and April 11, 2020. From 14 to 21 days following testing, we called patients to provide demographic and socioeconomic information and detailed exposure histories in the two weeks preceding illness onset. Respondent exposures and behaviors were described (specifying number missing among asked because of differences in denominators due to minor survey modifications after 3 days of piloting). Prior “close contact” with a case was defined as being physically within six feet. The study received a public health surveillance non‐research determination from Vanderbilt University and the Centers for Disease Control and Prevention.

Of 156 selected, 60% (93/156) completed the interview (89 patients and 4 proxy respondents). Fifty‐five percent (51/93) were female; median age was 41 years (IQR: 29‐53); and 57% (53/93), non‐Hispanic white; 15% (14/93), non‐Hispanic black; 13% (12/93), Hispanic; and 15% (14/93), other. Among respondents, 54% (49/90) reported close contact with somebody known to have COVID‐19 in the 2 weeks before illness onset (Table 1), most commonly a family member (47% [23/49]) or work colleague (47% [23/49]), some with multiple exposures. A majority reported being employed (78%, 70/90, 3 missing), with only 23% (16/69, 1 missing) able to telework. Regarding social distancing behaviors, only 12% (11/90, 3 missing) attended any gatherings with ≥10 people. Among those without an identified close case contact, 78% (32/41, 0 missing) were employed and 82% (23/28, 1 missing) of these worked outside of the home every day during the two weeks before illness, compared with 44% (14/32, 0 missing) of those with a known COVID‐19 contact. Although not systematically asked, nineteen respondents reported working at a local meat processing plant; of these, only 47% (9/19) reported having known close contact with a person infected with COVID‐19 before their illness.

**TABLE 1:** Self‐reported exposures for non‐hospitalized laboratory‐confirmed COVID‐19 patients during the two weeks prior to illness onset in Tennessee, March‐April 2020

Self‐reported characteristics and activities with possible influence on exposure risk in two weeks prior to illness	Overall^a^ N = 93	No close contact with known COVID‐19 case N = 41
Close contact with another COVID‐19 case	54% (49/90)	‐‐
Residence type		
Single‐family home	81% (75/93)	78% (32/41)
Apartment	15% (14/93)	17% (7/41)
Long‐term care facility	0% (0/93)	0% (0/41)
Group home	0% (0/93)	0% (0/41)
Other^b^	4% (4/93)	5% (2/41)
Lives with others	88% (82/93)	83% (34/41)
If lives with others, number in household	2 (1‐6)	2 (1‐6)
Average number daily contacts (within 6 feet)^c^	5 (0‐75)	5 (0‐75)
Employed	78% (70/90)	78% (32/41)
If employed, days working outside home		
Every day	62% (37/60)	82% (23/28)
2‐3 times a week	22% (13/60)	11% (3/28)
Once a week	0% (0/60)	0% (0/28)
Never	17% (10/60)	7% (2/28)
If employed, ability to telework	23% (16/69)	25% (8/32)
If employed, working in healthcare facility	21% (15/70)	16% (5/32)
Days interacting with others outside of home		
Every day	53% (40/76)	62% (23/37)
2‐3 times a week	22% (17/76)	19% (7/37)
Once a week	12% (9/76)	8% (3/37)
Never	13% (10/76)	11% (4/37)
Days going out for groceries		
Every day	0% (0/74)	0% (0/36)
2‐3 times a week	32% (24/74)	33% (12/36)
Once a week	38% (28/74)	44% (16/36)
Never	30% (22/74)	22% (8/36)
Attended gathering with 10 or more people	12% (11/90)	15% (6/41)
If yes, number of times past 14 days	2 (1‐14)	1.5 (1‐14)
Used public transportation	4% (4/90)	5% (2/41)
If yes, number of times past 14 days	9 (4‐24)	9 (4‐14)

^a^ Denominators vary for some exposures as additional questions were added after 3 d; results presented as % (No./Total No.) or median (range).

^b^ Others included condominium, hotel room, room in house, and refused to answer.

^c^ Maximum allowable number of daily contacts in survey was 75; interquartile range average of 2‐10 contacts/day overall and 2‐17 in those without a known COVID‐19 contact in the 2 wk before symptom onset.

In this cross‐sectional survey, we generated insights into individual‐level exposures and behaviors among non‐hospitalized adults with COVID‐19 in Tennessee in the early days following national social distancing guidelines. Notably, only half of respondents could identify a close contact with a COVID‐19 case in the two‐week period before illness onset. Transmission of SARS‐CoV‐2 is likely facilitated through a pre‐symptomatic or pauci‐symptomatic shedding period in infected individuals, ^2^, ^3^ which, along with limited early testing, could in part explain this common lack of a known case contact and highlights the need for robust testing infrastructure. Respondents reported engaging in practical social distancing behaviors such as avoiding large gatherings. However, a sizeable majority reported working regularly in the two weeks before illness with few able to telework, suggesting workplace‐related exposures were an important early source of transmission in Tennessee. As the United States returns to the workplace, these early findings underscore the need for ongoing workplace safety measures to prevent transmission. If teleworking is not feasible, workplaces should employ practices to reduce transmission including identifying where and how workers could be exposed, establishing organizational policies and practices for social distancing more than 6 feet, performing at‐work symptom screening, testing, and contact tracing once an infected employee is identified, and providing workplace education. ^4^ Case‐control studies should be considered to better understand workplace‐associated risks.

## CONFLICT OF INTEREST

This research was supported by the Centers for Disease Control and Prevention (contract number 75D30120C07637). CJL reports grants from National Institutes of Health, grants from Department of Defense, grants from Marcus Foundation, other from Endpoint Health, other from Entegrion, and other from Bioscape Digital, outside the submitted work. All other authors have no declarations.

## AUTHOR CONTRIBUTION

**Mark W Tenforde:** Conceptualization (equal); Formal analysis (lead); Investigation (equal); Methodology (equal); Supervision (equal); Writing‐original draft (lead). **Leora R Feldstein:** Conceptualization (equal); Formal analysis (supporting); Investigation (equal); Project administration (equal); Supervision (equal); Writing‐review & editing (equal). **Christopher J Lindsell:** Conceptualization (equal); Data curation (equal); Investigation (equal); Methodology (equal); Project administration (equal); Resources (equal); Writing‐review & editing (equal). **Manish M Patel:** Conceptualization (equal); Investigation (equal); Methodology (equal); Resources (equal); Supervision (equal); Writing‐review & editing (equal). **Wesley H Self:** Conceptualization (equal); Funding acquisition (equal); Investigation (equal); Methodology (equal); Resources (equal); Supervision (equal); Writing‐review & editing (equal).
